# Comparison of Mochi Needle and Conventional Hand Suturing for Laparoscopic Port Closure: A Prospective Comparative Study

**DOI:** 10.7759/cureus.110787

**Published:** 2026-06-13

**Authors:** Niroop Sri Raghava K N, Ravikiran H R

**Affiliations:** 1 General surgery, Sri Devaraj Urs Medical College, Kolar, IND; 2 General Surgery, Sri Devaraj Urs Medical College, Kolar, IND

**Keywords:** fascial closure, laparoscopic port closure, laparoscopic surgery, mochi needle, operative efficiency, port-site closure, postoperative complications, surgical technique, trocar-site closure, trocar-site hernia

## Abstract

Background

Secure laparoscopic port-site closure is essential to prevent postoperative complications such as wound infection, wound dehiscence, and trocar-site hernia, particularly at port sites measuring 10 mm or more. Conventional hand suturing remains the standard technique for fascial closure; however, it may be technically demanding and time-consuming, especially in patients with deep port sites or increased abdominal wall thickness. The Mochi needle is a reusable fascial closure instrument designed to facilitate controlled fascial approximation during laparoscopic port-site closure. This study aimed to compare Mochi needle closure and conventional hand suturing with respect to operative efficiency, technical feasibility, and short-term postoperative outcomes.

Methods

This prospective comparative study was conducted in the Department of General Surgery at R.L. Jalappa Hospital and Research Centre, Kolar, Karnataka, India, from March 2026 to May 2026, after obtaining Institutional Ethics Committee approval. A total of 100 patients undergoing elective laparoscopic procedures requiring closure of port sites measuring 10 mm or more were included. Patients were allocated into two groups of 50 each using an odd-even allocation method based on serial patient enrollment numbers. Group A underwent port-site closure using the Mochi needle technique, whereas Group B underwent conventional hand suturing using a standard round-body needle. The primary outcome measure was fascial closure time. Secondary outcomes included ease of procedure assessed using a five-point Likert scale, wound-related complications within seven postoperative days, the number of needle passes required for closure, and the need for additional intraoperative assistance during port-site closure.

Results

The mean fascial closure time was significantly shorter in the Mochi needle group compared to the conventional suturing group (2.6 ± 0.6 minutes vs. 5.3 ± 0.8 minutes; t = -19.10, p<0.001). Ease-of-procedure scores were significantly higher in the Mochi needle group (4.5 ± 0.5 vs. 3.7 ± 0.7; t = 6.58, p=0.002). The Mochi needle group also required fewer needle passes (2.1 ± 0.5 vs. 4.3 ± 0.9; p<0.001) and less additional intraoperative assistance during closure (4% vs. 18%; p=0.02). Wound-related complications were infrequent in both groups, with no statistically significant difference observed between the two techniques during the seven-day follow-up period.

Conclusion

The Mochi needle technique demonstrated significantly improved operative efficiency, fewer needle passes, and reduced requirement for additional assistance compared with conventional hand suturing for laparoscopic port-site closure. Short-term wound outcomes were comparable between the two groups. Further multicenter studies with larger sample sizes and longer follow-up are required to evaluate long-term outcomes, including trocar-site hernia incidence and overall clinical effectiveness.

## Introduction

Laparoscopic surgery has become an integral component of contemporary surgical practice because of its well-established advantages over open surgery, including reduced postoperative pain, shorter hospital stay, faster recovery, and improved cosmetic outcomes. With the increasing adoption of minimally invasive procedures, proper closure of laparoscopic port sites has become increasingly important in preventing complications such as wound infection, bleeding, wound dehiscence, chronic pain, and trocar-site hernia [1,2].

Trocar-site hernia is an uncommon but clinically significant complication following laparoscopic surgery [1]. Inadequate fascial closure of larger trocar sites has been identified as one of the major contributing factors for hernia formation. Trocar-site hernias may present with pain, bowel obstruction, incarceration, or strangulation and may occasionally require reoperation [1,3]. Therefore, secure fascial approximation during port-site closure remains an essential component of laparoscopic surgery and an important preventive measure against postoperative morbidity [2,4].

Conventional hand suturing remains the most widely practiced method for laparoscopic port-site closure because of its simplicity, availability, and familiarity among surgeons [2,3]. However, the technique may be technically demanding in obese individuals, patients with thick abdominal walls, and deep port sites. Difficulty in fascial visualization, repeated needle handling, limited access to the fascial plane, and increased tissue manipulation may prolong operative time and increase technical complexity during closure [3,5]. These challenges have encouraged the development of alternative closure techniques and devices aimed at improving operative efficiency and facilitating easier fascial approximation.

Various laparoscopic port-site closure methods have been described in the literature, including suture passer devices, fascial closure systems, Deschamps needles, Endo Close™ devices (Medtronic (formerly Covidien), Minneapolis, MN, USA), and modified closure needles [2-5]. Although many commercially available closure devices have demonstrated effectiveness, their routine use may be limited in certain settings because of increased cost, limited availability, or technical complexity. Consequently, simple and cost-effective alternatives that facilitate secure fascial closure may be particularly valuable in resource-limited healthcare settings.

The Mochi needle (Addler Laparoscopic Mochi Needle, Golden Nimbus India Pvt. Ltd., Mumbai, India) is a reusable stainless-steel fascial closure instrument with a curved pointed distal tip containing an eye for suture passage. It is designed to facilitate fascial approximation during laparoscopic port-site closure. Most available studies focus on trocar-site complications and general closure methods rather than direct comparison of operative efficiency and technical feasibility [2-5]. Further clinical evaluation is therefore necessary to assess the practicality and short-term outcomes associated with the Mochi needle technique in routine laparoscopic practice.

The present study was undertaken to compare Mochi needle closure and conventional hand suturing for laparoscopic port-site closure with respect to fascial closure time, ease of procedure, number of needle passes required for closure, need for additional intraoperative assistance, and short-term wound-related complications.

## Materials and methods

This prospective comparative study was conducted in the Department of General Surgery at the R.L. Jalappa Hospital and Research Centre, Sri Devaraj Urs Academy of Higher Education and Research, Kolar, Karnataka, India, between March 2026 and May 2026 after obtaining approval from the Central Ethics Committee of the institution (Approval No. SDUAHER/R&D/CEC/SDUMC-PG/402/NF/2025-26). Written informed consent was obtained from all participants prior to enrollment.

The sample size was calculated based on an expected difference in mean port-site fascial closure time between the two techniques, with a confidence level (CI) of 95% and a study power of 80%. A minimum sample size of 94 patients was obtained and rounded to 100 participants.

Patients aged 18-60 years undergoing elective laparoscopic procedures requiring closure of port sites measuring 10 mm or more were included in the study. Only patients with American Society of Anesthesiologists (ASA) physical status I or II [6] were considered eligible. Patients undergoing emergency laparoscopic procedures, those with significant comorbid conditions affecting wound healing, and those unwilling to provide informed consent were excluded.

A total of 100 eligible patients were enrolled and allocated into two groups of 50 each using an odd-even allocation method based on serial patient enrollment numbers. Patients with odd serial numbers were assigned to Group A and underwent laparoscopic port-site closure using the Mochi needle technique with absorbable polyglactin (Vicryl®, Ethicon, Inc., Somerville, NJ, USA) sutures, whereas patients with even serial numbers were assigned to Group B and underwent conventional hand suturing using a standard round-body needle and the same suture material. The majority of patients underwent laparoscopic cholecystectomy (49%) and laparoscopic appendectomy (47%), while diagnostic laparoscopy and laparoscopic hernia repair each accounted for 2% of cases.

All procedures were performed under general anesthesia using standard laparoscopic techniques. Port-site fascial closure was performed by third-year postgraduate surgical residents under consultant supervision. All participating residents had prior exposure to basic laparoscopic procedures and port-site closure techniques through routine surgical training. Standard aseptic precautions were followed in all cases.

**Figure 1:**
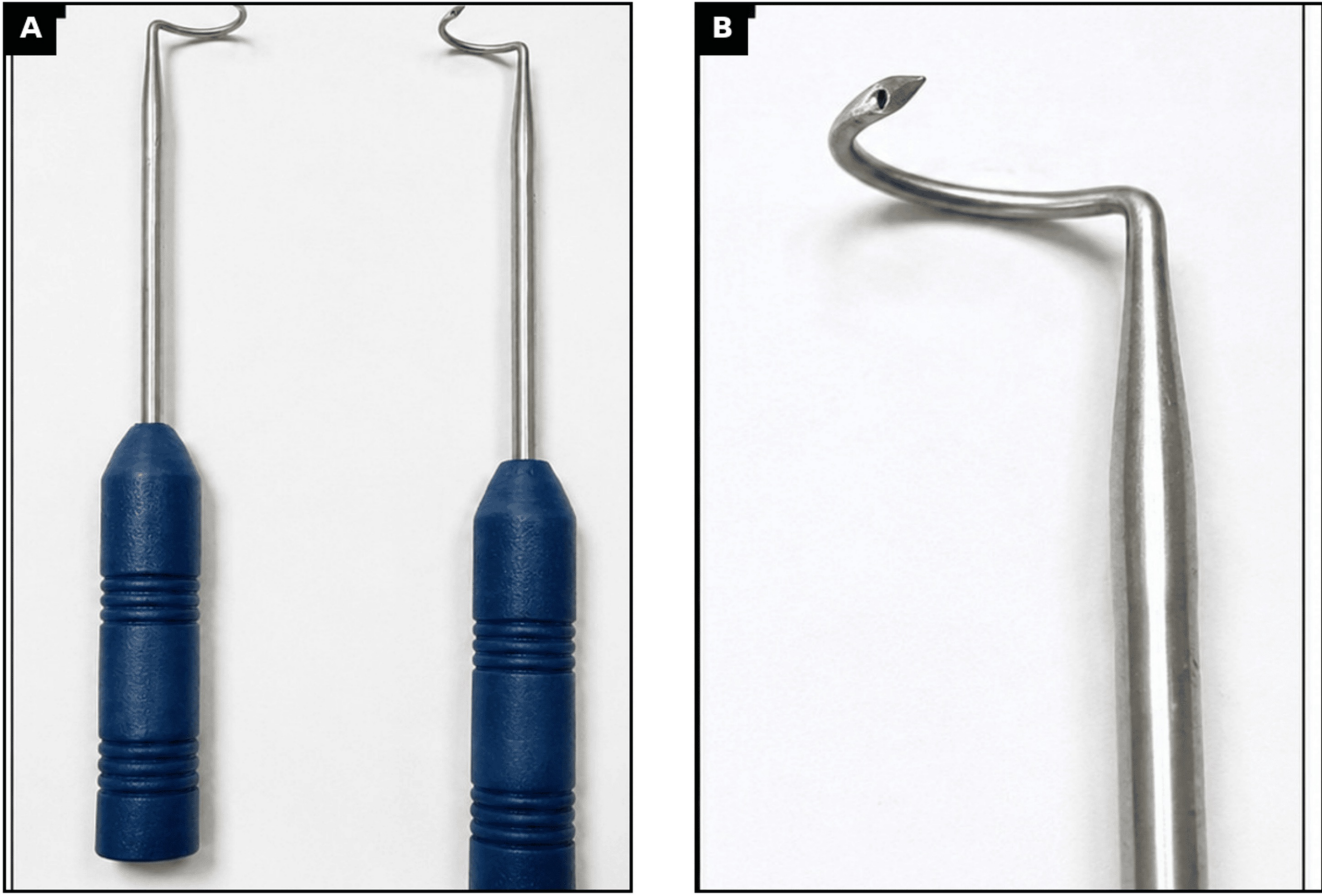
Mochi needle used for laparoscopic port-site closure (A) Mochi needle; (B) Close-up view of the curved distal tip with an eye for suture passage and retrieval.

The Mochi needle is a reusable stainless-steel fascial closure instrument consisting of a straight shaft with a curved pointed distal tip containing an eye for suture passage as shown in Figure 1.

**Figure 2:**
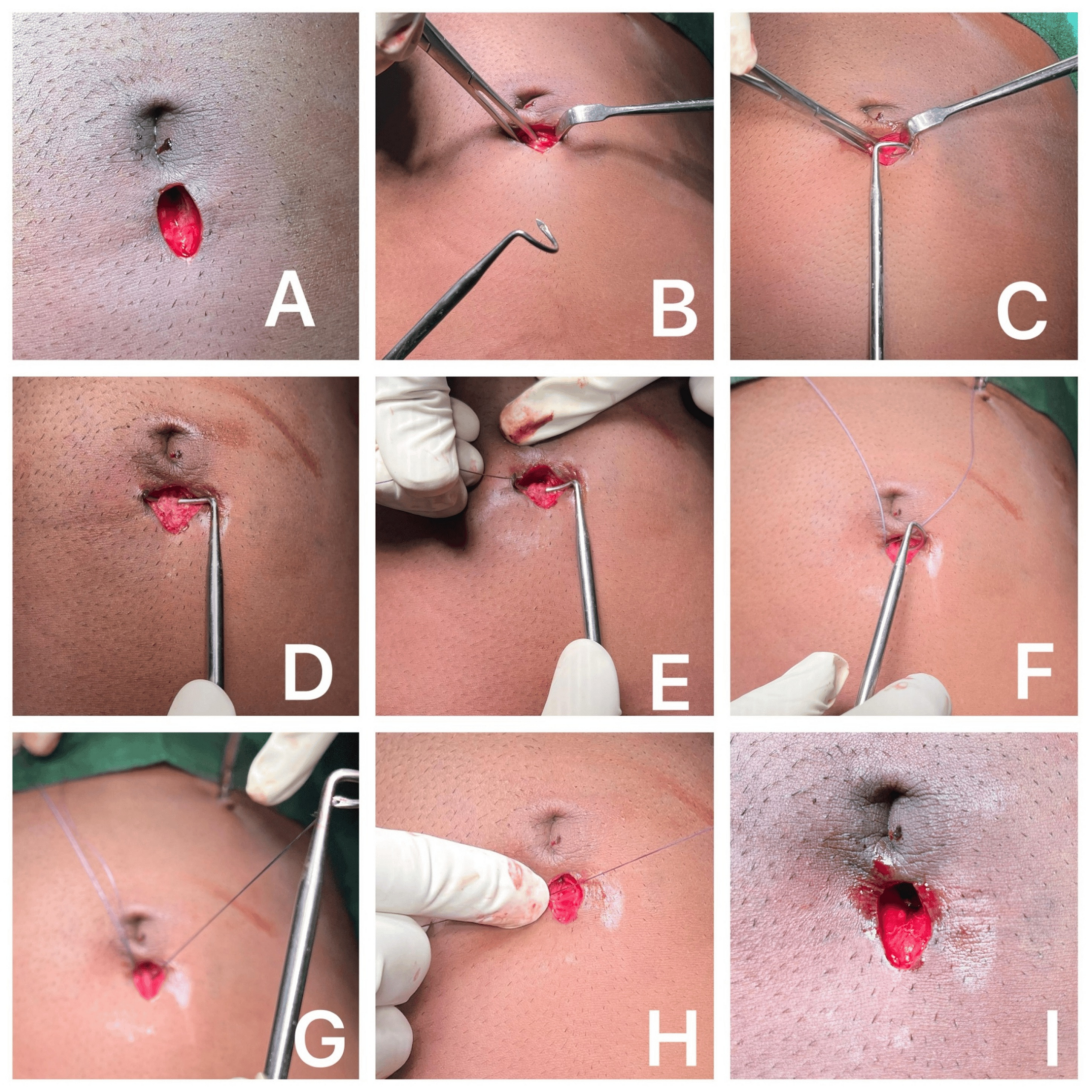
Sequential steps of the Mochi needle technique for laparoscopic port-site closure (A) Port-site fascial defect after trocar removal; (B) Introduction of the Mochi needle at one edge of the fascial defect; (C) Passage of the needle through the fascial layers; (D) Advancement of the needle beneath the fascial defect; (E) Retrieval of the needle through the opposite fascial edge; (F) Passage of the suture through the eye of the needle; (G) Withdrawal of the needle carrying the suture through the fascial tract; (H) Approximation of the fascial edges following suture retrieval. (I) Final appearance of the fascial defect after completion of the suture passage prior to knot tying.

Following removal of the laparoscopic port, the fascial defect was identified. The Mochi needle was introduced through one fascial-muscular edge and advanced through the posterior fascial layer. The needle was then directed beneath the defect and passed through the opposite fascial-muscular edge, allowing the eye of the needle to emerge externally. An absorbable polyglactin (Vicryl) suture was threaded through the eye of the needle, following which the needle was withdrawn along the same path through both fascial bites. The fascial edges were then approximated and secured with knot tying. Representative intraoperative photographs demonstrating the sequential steps of the technique are shown in Figure 2.

The primary outcome measure was port-site fascial closure time, defined as the time measured in minutes from insertion of the needle for the first fascial bite until completion of the final knot securing fascial closure. Closure time was recorded using a stopwatch by an independent observer.

Secondary outcome measures included ease of procedure, wound-related complications, number of needle passes required for fascial closure, and need for additional intraoperative assistance during closure. Ease of port-site closure was assessed immediately after completion of closure by the operating resident using a five-point Likert scale ranging from one (very difficult) to five (very easy). Wound-related complications were assessed clinically within seven postoperative days and included signs of port-site infection such as redness, tenderness, discharge, or wound gaping. The number of needle passes required for successful fascial closure was recorded intraoperatively. Additional intraoperative assistance was defined as the need for consultant assistance, additional fascial exposure, or use of supplementary instruments during closure.

Data were entered into Microsoft Excel (Microsoft Corp., Redmond, WA, USA) and analyzed using IBM SPSS Statistics for Windows, Version 26 (Released 2019; IBM Corp., Armonk, New York, United States). Continuous variables were expressed as mean ± standard deviation and compared using Student's t-test, whereas categorical variables were expressed as frequencies and percentages and analyzed using the chi-square test or Fisher's exact test where appropriate. Test statistic values along with p-values were reported for all comparisons. A p-value of less than 0.05 was considered statistically significant.

## Results

A total of 100 patients undergoing elective laparoscopic surgery were included in the study, with 50 patients allocated to the Mochi needle group and 50 patients allocated to the conventional hand suturing group. Both groups were comparable with respect to demographic and baseline clinical characteristics. No statistically significant differences were observed between the two groups, with regard to age, sex distribution, ASA physical status, or body mass index, indicating comparable baseline characteristics (Table 2).

**Table 1:** Baseline characteristics of the study population (n=100) Baseline characteristics of the study population. Continuous variables were analyzed using Student’s t-test, whereas categorical variables were analyzed using the chi-square test. ASA: American Society of Anesthesiologists.

Variable	Mochi needle group (n=50)	Conventional suturing group (n=50)	Statistical test (t/χ²)	p-value
Mean age (years)	41.2 ± 9.1	42.5 ± 8.7	t = -0.73	0.48
Male/Female	28/22	30/20	χ² = 0.17	0.68
ASA I/II	32/18	30/20	χ² = 0.18	0.67
Mean BMI (kg/m²)	24.8 ± 3.2	25.1 ± 3.4	t = -0.45	0.65

The study population predominantly comprised patients undergoing laparoscopic cholecystectomy and laparoscopic appendectomy, which together accounted for 96% of all procedures performed (Table 2).

**Table 2:** Distribution of laparoscopic procedures in the study population (n=100)

Procedure	Number (%)
Laparoscopic cholecystectomy	49 (49%)
Laparoscopic appendectomy	47 (47%)
Diagnostic laparoscopy	2 (2%)
Laparoscopic hernia repair	2 (2%)

The mean port-site fascial closure time was significantly shorter in the Mochi needle group compared with the conventional suturing group (2.6 ± 0.6 minutes versus 5.3 ± 0.8 minutes; t = -19.10, p<0.001). Ease-of-procedure scores assessed using a five-point Likert scale were significantly higher in the Mochi needle group (4.5 ± 0.5 versus 3.7 ± 0.7; t=6.58, p=0.002), indicating improved perceived technical convenience during closure (Table 3).

**Table 3:** Comparison of operative and technical outcome measures in the study population (n=100) Continuous variables were analyzed using Student's t-test. Categorical variables were analyzed using the chi-square test.

Outcome measure	Mochi needle group (n=50)	Conventional suturing group (n=50)	Statistical test (t/χ²)	p-value
Closure time (minutes)	2.6 ± 0.6	5.3 ± 0.8	t = -19.10	<0.001
Ease of procedure score	4.5 ± 0.5	3.7 ± 0.7	t = 6.58	0.002
Needle passes required	2.1 ± 0.5	4.3 ± 0.9	t = -15.72	<0.001
Additional assistance required	2 (4%)	9 (18%)	χ² = 5.01	0.02

The mean number of needle passes required for fascial closure was significantly lower in the Mochi needle group than in the conventional suturing group (2.1 ± 0.5 versus 4.3 ± 0.9; t = -15.72, p<0.001). Additional intraoperative assistance during closure was also required less frequently in the Mochi needle group, with only two patients (4%) requiring assistance compared with nine patients (18%) in the conventional suturing group (χ² = 5.01, p=0.02).

As ease-of-procedure scores were based on operator assessment, this outcome remains inherently subjective.

Wound-related complications were minimal in both groups. One patient (2%) in the conventional suturing group developed a superficial port-site infection within seven postoperative days, whereas no wound infections were observed in the Mochi needle group. No cases of wound gaping were observed in either group. The difference in wound-related complications between the two techniques was not statistically significant (Table 4).

**Table 4:** Wound-related complications within seven postoperative days Categorical variables were analyzed using Fisher’s exact test where appropriate.

Complication	Mochi needle group (n=50)	Conventional suturing group (n=50)	Statistical test	p-value
Port-site infection	0 (0%)	1 (2%)	Fisher’s Exact Test	0.31
Wound gaping	0 (0%)	0 (0%)	—	—

Overall, the Mochi needle technique demonstrated significantly improved operative efficiency and procedural convenience, with fewer needle passes and reduced need for additional assistance, while maintaining comparable short-term wound outcomes. However, the study was not designed or powered to assess long-term complications such as trocar-site hernia formation.

## Discussion

Secure fascial closure of laparoscopic port sites measuring 10 mm or greater remains an important component of minimally invasive surgery because inadequate closure has been associated with wound complications and trocar-site hernia formation [1,2]. Although conventional hand suturing remains the most widely practiced method of port-site closure, the technique may be technically challenging in patients with deep port sites, thick abdominal walls, or limited fascial visualization [3]. The present study evaluated the Mochi needle technique as an alternative method for laparoscopic port-site closure and demonstrated significant improvements in operative efficiency and technical convenience compared with conventional hand suturing.

The primary finding of this study was the significant reduction in fascial closure time achieved with the Mochi needle technique. The mean closure time was reduced from 5.3 ± 0.8 minutes in the conventional suturing group to 2.6 ± 0.6 minutes in the Mochi needle group. This improvement may be attributed to the design of the Mochi needle, which facilitates controlled fascial capture and suture retrieval while minimizing repeated needle manipulations. Several reviews have highlighted the importance of secure and efficient fascial closure techniques to reduce port-site complications following laparoscopic surgery [2-5]. Although the absolute reduction in closure time was approximately 2.7 minutes per port site, this difference may be clinically relevant in high-volume surgical units and procedures requiring multiple port closures, potentially improving operating room efficiency and workflow.

The Mochi needle technique also demonstrated superior technical feasibility. Ease-of-procedure scores were significantly higher, while the number of needle passes required for successful fascial closure was significantly lower compared with conventional hand suturing. Fewer needle passes may reduce tissue manipulation and facilitate closure in patients with deeper abdominal walls or difficult fascial access. Various alternative fascial closure techniques and devices have been described in the literature to facilitate secure port-site closure and reduce technical difficulty [4,5,7]. However, ease-of-procedure scores were based on operator assessment and should therefore be interpreted as subjective measures of perceived technical convenience.

An additional finding was the significantly lower requirement for intraoperative assistance in the Mochi needle group. Conventional fascial closure often requires additional retraction or assistance to improve visualization of the fascial defect, particularly in technically challenging cases. Reduced dependence on assistance may improve workflow efficiency and may be particularly useful in resource-constrained settings and teaching institutions where operative efficiency is important.

Short-term wound-related complications were infrequent in both groups. No statistically significant differences were observed with respect to port-site infection or wound gaping. These findings suggest that the Mochi needle technique provides effective short-term fascial approximation comparable to conventional hand suturing. However, the present study was not designed or powered to detect differences in uncommon complications. Furthermore, follow-up was limited to seven postoperative days and therefore did not permit assessment of long-term outcomes such as trocar-site hernia formation, which remains the most clinically relevant complication following laparoscopic port-site closure [1-4]. Consequently, the absence of significant differences in early wound complications should not be interpreted as evidence of long-term equivalence between the two techniques.

The strengths of the present study include its prospective design, clearly defined outcome measures, and direct comparison of two clinically relevant fascial closure techniques. Objective assessment of closure time by an independent observer further strengthened the reliability of the primary outcome measure. In addition, the inclusion of operative photographs and a detailed description of the Mochi needle technique improves reproducibility and facilitates future evaluation by other surgeons.

Several limitations should be considered when interpreting these findings. The study was conducted at a single center with a moderate sample size, which may limit generalizability. Patient allocation was performed using an odd-even allocation method rather than concealed randomization, introducing the possibility of allocation bias. Fascial closure was performed by third-year postgraduate surgical residents under consultant supervision, introducing potential operator-dependent variability. Ease-of-procedure assessment was subjective and unblinded. Furthermore, follow-up was limited to seven postoperative days and therefore did not permit assessment of long-term trocar-site hernia incidence. Larger multicenter studies with longer follow-up durations are required to validate these findings and determine the long-term clinical effectiveness of the Mochi needle technique.

## Conclusions

The Mochi needle technique significantly reduced fascial closure time, needle passes, and the requirement for additional intraoperative assistance compared with conventional hand suturing. Ease-of-procedure scores were also higher, indicating improved perceived technical convenience during laparoscopic port-site closure. Short-term wound-related outcomes were comparable between the two groups, with no increase in early postoperative complications observed. These findings suggest that the Mochi needle is an efficient and practical alternative for laparoscopic port-site closure. However, the present study was limited to short-term follow-up and was not designed to evaluate long-term complications such as trocar-site hernia formation. Larger multicenter studies with extended follow-up are required to confirm these findings and establish long-term clinical effectiveness.
